# Practitioner and scientist perceptions of successful amphibian conservation

**DOI:** 10.1111/cobi.13005

**Published:** 2018-01-10

**Authors:** Helen M.R. Meredith, Freya A.V. St. John, Ben Collen, Simon A. Black, Richard A. Griffiths

**Affiliations:** ^1^ Durrell Institute of Conservation and Ecology, School of Anthropology and Conservation University of Kent Canterbury CT2 7NR U.K.; ^2^ Institute of Zoology Zoological Society of London Regent's Park London NW1 4RY U.K.; ^3^ Centre for Biodiversity and Environment Research, Department of Genetics, Evolution and Environment University College London Gower Street London WC1E 6BT U.K.; ^4^ Current address: Amphibian Survival Alliance Synchronicity Earth 32A Thurloe Place London SW7 2HQ U.K.; ^5^ Current address: School of Environment, Natural Resources and Geography Bangor University Bangor, Gwynedd LL57 2UW U.K.

**Keywords:** amphibian declines, caecilian, evaluation, frog, inventory and monitoring, salamander, Cecilia, declinación de anfibios, evaluación, inventario y monitoreo, rana, salamandra

## Abstract

Conservation requires successful outcomes. However, success is perceived in many different ways depending on the desired outcome. Through a questionnaire survey, we examined perceptions of success among 355 scientists and practitioners working on amphibian conservation from over 150 organizations in more than 50 countries. We also sought to identify how different types of conservation actions and respondent experience and background influenced perceptions. Respondents identified 4 types of success: species and habitat improvements (84% of respondents); effective program management (36%); outreach initiatives such as education and public engagement (25%); and the application of science‐based conservation (15%). The most significant factor influencing overall perceived success was reducing threats. Capacity building was rated least important. Perceptions were influenced by experience, professional affiliation, involvement in conservation practice, and country of residence. More experienced practitioners associated success with improvements to species and habitats and less so with education and engagement initiatives. Although science‐based conservation was rated as important, this factor declined in importance as the number of programs a respondent participated in increased, particularly among those from less economically developed countries. The ultimate measure of conservation success—population recovery—may be difficult to measure in many amphibians; difficult to relate to the conservation actions intended to drive it; and difficult to achieve within conventional funding time frames. The relaunched Amphibian Conservation Action Plan provides a framework for capturing lower level processes and outcomes, identifying gaps, and measuring progress.

## Abstract 2

*Article impact statement*: Conservation scientists and practitioners perceive success differently; thus, a clear definition of *success* up front improves results.

## Abstract (es)

Percepciones de los Científicos y los Practicantes sobre la Conservación Exitosa de Anfibios

**Resumen**

La conservación requiere de resultados exitosos. Sin embargo, el éxito se percibe de diferentes maneras dependiendo del resultado deseado. A través de una encuesta, examinamos la percepción del éxito entre 355 científicos y practicantes que trabajan en la conservación de anfibios en más de 150 organizaciones de más de 50 países. También buscamos identificar cómo los diferentes tipos de acciones y la experiencia de los respondientes influyen sobre las percepciones. Los respondientes identificaron cuatro tipos de resultados exitosos: mejoras para las especies y el hábitat (84% de los respondientes), manejo efectivo del programa (36%), iniciativas de alcance como la educación y la participación pública (25%), y la aplicación de la conservación basada en la ciencia (15%). El factor más significativo que influye sobre el éxito percibido general fue la reducción de amenazas. La creación de capacidad fue valorada como el menos importante. Las percepciones estuvieron influenciadas por la experiencia, la afiliación profesional, la participación en la práctica de la conservación, y el país de residencia. Los practicantes con mayor experiencia asociaron más al éxito con las mejoras para las especies y los hábitats y menos con la educación y las iniciativas de compromiso. Aunque la conservación basada en la ciencia estuvo valorada como importante, este factor declinó en importancia conforme el número de programas en los que participaron los respondientes incrementó, particularmente entre aquellos que viven en países con menor desarrollo económico. La medida definitiva del éxito de la conservación – la recuperación de la población – puede que sea difícil de medir en muchos anfibios; difícil de relacionar con las acciones de conservación que pretenden conducirla; y difícil de conseguir dentro de los marcos convencionales de tiempo de financiamiento. El Plan de Acción de Conservación de Anfibios lanzado nuevamente proporciona un marco de trabajo para capturar resultados y procesos de niveles más bajos, identificar vacíos y medir el progreso.

## Introduction

Although the roots of conservation biology can be traced back over many decades, the field emerged as a scientific discipline over 30 years ago (Soulé 1985) and continues to evolve (Kareiva & Marvier 2012). Those aspects of conservation associated with success (hereafter perceptions of success) are also evolving, which leads to different views on what comprises success. Despite considerable global efforts to conserve biological diversity (Rands et al. 2010), conservation success is rarely defined, measured, and communicated (Saterson et al. 2004). The purpose of conservation may be framed in multiple ways that affect the measurement of success (Mace 2014). Uncertainty in defining success can confound efforts to assess the value and relative level of achievement of conservation projects, and in conservation the diversity of definitions of success (e.g., Kleiman et al. 2000; Young et al. 2014) can cause confusion in assigning goals, and vice versa.

Nature is increasingly valued in terms of ecosystem services that benefit people (Mace 2014), emphasizing conservation achievement alongside enhanced human well‐being (Carpenter et al. 2009; Ostrom 2009; Kapos et al. 2010). Community‐based conservation projects (as classified by Souto et al. [2014]) associate success with supportive social processes that encompass the needs, values, and awareness of local stakeholders and the general public (Clark & Wallace 1998; Mascia et al. 2003), such as development of sustainable livelihoods and improved welfare of local stakeholders (du Toit et al. 2004; Davies et al. 2014). This anthropocentric focus on measuring conservation success has been dubbed the “new conservation” and entails replacing species and habitat interventions with economic development and poverty reduction (Soulé 2013).

Conservation success relates to the impact of different conservation actions (Kapos et al., 2008, 2009, 2010). These include measures of process (e.g., species and site management, capacity building, political lobbying, financial indicators, resource utilization, milestones, research, learning by local communities, and operational capability) and measures of purpose‐related outcome (e.g., population recovery, recovered habitats, sustained support in local communities, legal statutes, sustained human benefits, and poverty alleviation). If these are not aligned, then an organization might, for example, achieve research goals at the expense of local support or to the detriment of nontarget species. Alternatively, the program may operate smoothly, yet fail to deliver its desired outcomes, as observed in many instances (Kleiman et al. 2000; Black & Groombridge 2010; Black et al. 2011; Martin et al. 2012).

We explored the perceptions of success held by amphibian conservation scientists and practitioners. Our aim was to investigate the range of views on the nature of success and the factors that may influence different perceptions. Amphibians are a large and widespread group in significant decline (Stuart et al., 2004, 2008). They are also the subject of concerted and long‐term conservation efforts (e.g., Griffiths & Pavajeau 2008; Smith & Sutherland 2014; Young et al. 2014), and there is a substantial group of practitioners and scientists focusing on their conservation (AArk 2016; ASA 2016; ASG 2016).

## Methods

### Data Collection

We interviewed 5 key informants engaged in a range of amphibian conservation activities at the 2012 Amphibian Conservation Research Symposium. Subsequently, we developed a pilot questionnaire based on these interviews and disseminated it among delegates of the 15th African Amphibian Working Group meeting in 2012. Pilot data informed the revision and improvement of the questions included in the final questionnaire. Hard copies of the questionnaire (Supporting Information) were distributed to respondents at the 7th World Congress of Herpetology (7WCH; August 2012). An identical questionnaire was disseminated to the IUCN SSC Amphibian Specialist Group online (http://www.surveymonkey.com; available August 2012–February 2013). We used a targeted sampling strategy to select potential respondents with relevant expertise and encouraged chain‐referral sampling (Newing 2011) to maximize sample size and breadth of respondents. Respondents were asked to provide details relating to 5 explanatory variables: their institution type; country of residence; whether they identified themselves as a conservation practitioner; whether or not they also conducted research; number of years of experience in conservation science or practice or both science and practice; and number of ongoing conservation programs (see Supporting Information for all definitions).

### Measuring Perceptions of Success

We initially asked respondents: “How do you perceive ‘success’ in a conservation programme?” (hereafter open‐ended question [question 12; Supporting Information]). We subsequently coded answers to permit quantitative assessment (Newing 2011). Using a 5‐point ordinal scale (1, not important, to 5, highly important; 0, not applicable), respondents then scored a series of statements describing aspects of perceived success in conservation (hereafter components [question 15; Supporting Information]) (Table 1). These are categorized in Kapos et al. (2008, 2009, 2010) as species and site management, sustainable resource use, education and awareness, capacity building, research, and government policy. From the same list, respondents then picked their top 3 statements, thus providing a measure of popularity. Permission to conduct this study was granted through ethical reviews from the 7WCH and the University of Kent.

**Table 1:** Statements of conservation success ordered by the percentage of respondents choosing the statement as one of their top 3 that best describe success (popularity)

Components of conservation success	Popularity (%)	Mean score (SE)*
Species and site management: reducing known threats to improve the response of conservation target species to conservation interventions	84	4.70 (0.04)
Research: applying appropriate research results to conservation practice	53	4.51 (0.05)
Sustainable resource use: promoting sustainable resource use and minimizing damaging practices by relevant stakeholders	47	4.26 (0.06)
Education and awareness: increasing support for the conservation of a species among appropriate target audience through a communication, education, and public‐awareness strategy	46	4.30 (0.06)
Government policy: implementing relevant policies or promoting legislation relevant to conservation aims	38	4.18 (0.06)
Capacity building: increasing the quality and/or quantity of conservation action(s) through appropriate capacity building (training of project staff)	32	4.09 (0.07)

*Mean scores of importance are out of a maximum of 5 from 1, not important in describing conservation success, to 5, highly important in describing conservation success (*n* = 245).

### Data Analyses

We analyzed data with R version 2.14.2 (R Core Team 2012), and all analyses preserved the anonymity of respondents. Answers to the open‐ended question were coded by dividing each full answer into a series of segments that noted discrete aspects of success (hereafter points). Each point was coded according to a defined list assembled after data collection (Newing 2011), and codes were allocated between 4 major categories: species and habitat points described direct improvements in species populations or habitats resulting from in situ or ex situ conservation interventions; program‐management points related to general program structure, management, and strategy; education and engagement points included public education and awareness activities and fostering local community or stakeholder support and involvement; and research and evaluation points addressed species and habitat‐related scientific research needs or the evaluation of program outcomes.

The proportion of points made by each respondent in each category was calculated by dividing the number of points in a category by the total number of points made across all categories. The proportion of responses for each of the 4 main categories was modeled separately as a function of 5 discrete explanatory variables: institution (academic or nonacademic); country (less economically developed countries [LEDCs] or more economically developed countries [MEDCs] [IMF 2014]); conservation practitioner (involved in practical conservation activities: yes or no); experience (in years, encompassing conservation science or practice); and conservation programs (number ongoing). We modeled each variable and all 2‐way interactions with generalized linear models (GLMs) with binomial error structure. A quasi‐binomial error distribution was employed when models were overdispersed (Crawley 2007). Starting with 2‐way interactions, models were simplified by removing the least significant factor. The resulting model was compared to the previous one with an *F* test (quasi‐binomial) or chi‐square test (binomial) before factor deletion. If the variance explained by the model before and after removal was significantly different, the interaction or variable was retained (Crawley 2007). The final model was accepted when only significant factors remained.

We analyzed importance scores given by respondents (0–5 scale) with GLM to investigate the perceptions of different components, namely, sustainable resource use, education and awareness; capacity building, research, and government policy (derived from Kapos et al. 2010). Per statement, each score was converted to a proportion of the maximum score (i.e., 5) with the initial model structure and simplification as above. We did not include the species and site management statement in this analysis because known threats could subsume aspects of the other components (e.g., unsustainable resource use could constitute a threat requiring management). For all analyses, effect sizes for explanatory variables of interest are presented in addition to their significance values. Effect sizes were calculated using Nagelkerke's pseudo *R* ^2^ (Nagelkerke 1991) and are interpreted according to Cohen's (1988) rule of thumb (0.1, small; 0.3, medium; 0.5, large).

## Results

### Questionnaire Responses

The questionnaire was answered by 355 respondents: 96 completed a paper questionnaire and 259 completed an online questionnaire. The 7WCH sample had a higher proportion of respondents from academic institutions (7WCH 60%; online 51%), and the online questionnaire attracted a greater proportion of respondents from LEDC countries (7WCH 11%; online 30%). Overall, the questionnaire was answered by 89 LEDC‐based respondents and 265 MEDC‐based respondents (one respondent did not report country of residence) across 55 countries and 167 organizations. The online questionnaire attracted proportionally more conservation practitioners (7WCH 38%; online 44%). Median years of experience were similar across the 2 samples: 6.5 years for 7WCH respondents (interquartile range [IQR], 4–19; *n =* 96) and 10 years for the online questionnaire (IQR 6–20; *n =* 259). The median number of conservation programs per respondent was one for both the 7WCH (IQR 0–3; *n =* 96) and online sample (IQR 0–2; range = 0–15; *n =* 259). The 2 sets of questionnaires were analyzed as a single sample to ensure the largest possible range of respondents.

### Perceptions of Success

The number of discrete points describing success in amphibian conservation ranged from 1 to 9 per respondent; 242 respondents assigned a total of 579 points. Responses described 19 different types of success covering both process and outcome measures (details given in Supporting Information) allocated among 4 categories: species and habitat (84%); program management (35%); education and engagement (24%); and research and evaluation (14%).

The majority of species and habitat points (96%; 349 points from 203 respondents) referred to in situ species conservation improvements (e.g., population numbers, persistence, security, genetic diversity, and health) and their habitats (e.g., condition, size, connectivity, and protection). The remaining 4% described ex situ conservation measures, whereby assurance colonies of species are maintained in captivity, especially in cases where in situ threats cannot currently be mitigated. Seventy‐six percent of program management points (113 points from 85 respondents) referred to considerations such as long‐term funding, multistakeholder approaches, clear strategic planning, an adaptive‐learning program framework, and effective personnel management, all of which relate to process. The remaining 24% of program management points asserted that success equals the achievement of predetermined program goals. Education and engagement points (77 points from 59 respondents) described public education and awareness initiatives (57%) or the development of local support, sustainable livelihoods, and local community or stakeholder involvement (43%). Research and evaluation points (40 points from 35 respondents) mentioned scientific research on species and habitat as being crucial to successful conservation (63%), as well as the evaluation of program outcomes through appropriate monitoring (37%).

Across the statements, the mean importance score out of 5 varied little (4.09–4.70) and mirrored the trend in the proportion of respondents selecting statements as being among their top 3, which exhibited a wider range. Species and site management was the most popular statement (84% of respondents chose among top 3 statements), and capacity building was the least popular (32%).

### Predictors and Components of Success

**Figure 1:**
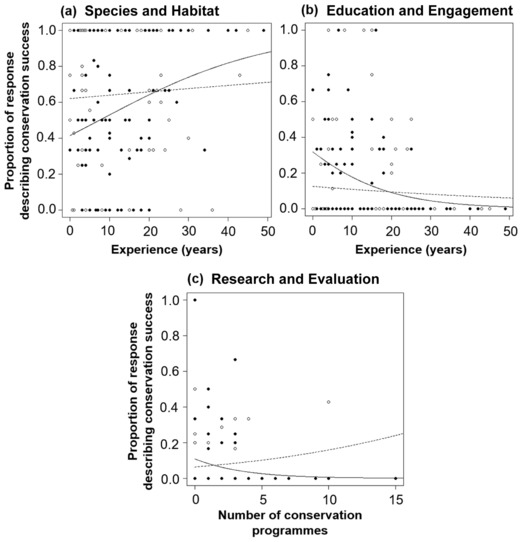
The relationship between types of conservation success ([a] species and habitat, [b] education and engagement, and [c] research and evaluation) noted by respondents (n = 242) and the explanatory variables of (a, b) extent of respondent experience in amphibian research and conservation practice (solid circles and lines, conservation practitioners; open circles, dashed lines, nonpractitioner) and (c) number of respondent's ongoing conservation programs. Fitted lines are model predictions of the change in the response variable (y‐axis) when all explanatory variables (x‐axis) in the final simplified models are held at their mean values.

Conservation practitioners believed species and habitat improvements to be proportionally less significant in defining conservation success than nonpractitioners. A significant interaction between the explanatory variables of conservation practitioner and experience (GLM: *t* = 2.0, SE 0.02, *p* = 0.05, df = 241, *R* ^2^ = 0.023) suggested that more experienced conservation practitioners believed factors relating to species and habitat were more important than less experienced practitioners (Fig. 1), although the effect size of this interaction was small. Conservation practitioners also considered education and engagement more important in defining conservation success than nonpractitioners. A significant interaction between the explanatory variables of conservation practitioner and experience and the importance of education and engagement in defining success (GLM: *t* = –2.0, SE 0.03, *p* = 0.04, df = 241, *R* ^2^ = 0.026) suggested a declining importance attributed to education and engagement by practitioners as experience increased (Fig. 1), but again the effect size for the interaction was small. In both cases (species and habitat and education and engagement), the perceptions of nonpractitioners altered little with experience (Fig. 1). The proportion of points relating to research and evaluation was low. However, academic respondents involved in multiple conservation programs assigned a greater proportion of points relating to research and evaluation than those based at nonacademic institutions. The importance of this category increased with the number of conservation programs for individuals from academic institutions but decreased to zero for respondents based at nonacademic institutions (GLM: *z* = –2.0, SE 0.02, *p* = 0.043, df = 241, *R* ^2^ = 0.056) (Fig. 1). No significant relationships were found between any of the interactions or discrete explanatory variables and the proportion of program management points made by respondents.

**Figure 2:**
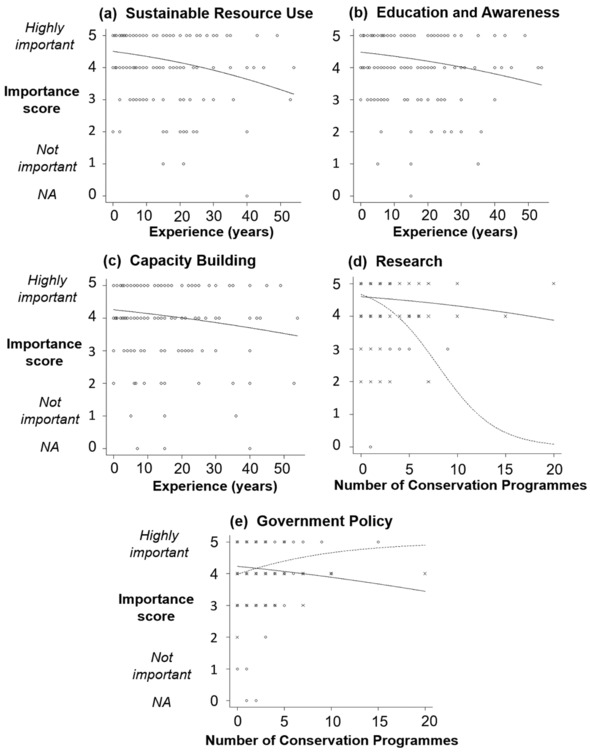
The relationship between respondent scores of importance for components of conservation success (5, highly important; 1, not important; NA, not applicable; n = 235) and (a, b, c) extent of respondent experience in amphibian research and conservation practice and (d, e) number of respondent's ongoing conservation programs (see Table 1 for descriptions of components): (a) sustainable resource use, (b) education and awareness, (c) capacity building, (d) research (open circles, dashed line, less economically developed countries; crosses, solid line, more economically developed countries); and (e) government policy (open circles, dashed line, nonacademic; crosses, solid line, academic). Fitted lines are model predictions of the change in the response variable (y‐axis) when all explanatory variables (x‐axis) in the final simplified models are held at their mean values.

Scores for components associated with investing in the human aspects of a conservation program (sustainable resource use, education and awareness, and capacity building; i.e., the components most analogous to the category education and engagement) were negatively related to years of experience across all respondents: sustainable resource use (GLM: *t* = –3.9, SE 0.008, *p* = <0.001, df = 234, *R* ^2^ = 0.103); education and awareness (GLM: *t* = –3.1, SE 0.008, *p* = 0.002, df = 234, *R* ^2^ = 0.068); and capacity building (GLM: *t* = –2.3, SE 0.008, *p* = 0.02, df = 234, *R* ^2^ = 0.038 (Fig. 2). In each case, the effect size of experience on response scores was small. For research, a significant interaction was found between country and conservation programs; the importance of research declined as the number of conservation programs per person increased and this decline was particularly pronounced for those from LEDC (GLM: *t* = 2.5, SE 0.11, *p* = 0.02, df = 234, *R* ^2^ = 0.046 (Fig. 2). Government‐policy scores were associated with a significant interaction between institution and conservation programs (*t* = 2.2, SE 0.08, *p* = 0.03, df = 234, *r* ^2^ = 0.034). As the number of programs increased, scores increased for respondents from nonacademic institutions but declined for those from academic institutions (Fig. 2).

## Discussion

### Perceptions of Success

Our results indicated a diversity of perceptions of success that were influenced by factors concerning the respondent's background. Previous researchers have similarly recognized that conservation success comes in different forms (Brooks et al. 2006; Waylen et al. 2010), at different spatial scales (Sodhi et al. 2011), and at different organizational levels (Mace et al. 2007). The achievement of in situ improvements in the status of species and habitats is overwhelmingly perceived as central to success in amphibian conservation. Ex situ conservation actions are advocated for amphibians when threat mitigation is impossible (Zippel et al. 2011), although they may not be suitable for all species (Tapley et al. 2015). Although ex situ measures can be crucial to averting extinctions (e.g., Lee et al. 2006), they may not be associated with long‐term success unless populations are restored to the wild.

Program management definitions of perceived success were related to effective organization of financial and human resources and the achievement of prestated goals. The sustained mobilization of financial and technical resources (McCarthy et al. 2012), effective leadership (Williams et al. 2007; Black et al. 2011; Walls et al. 2017), and the use of adaptive management and organizational learning (Clark 1996) are all associated with success, particularly as conservation programs become more interdisciplinary (Black & Copsey 2014; Pooley et al. 2014). Black and Groombridge (2010) investigated organizational measures of success in business management and adapted them to conservation projects. The implementation strategy of a conservation program is as crucial to its operational success as any of its component actions (Knight 2006).

Education and engagement can unite conservation with improvements in human welfare and livelihoods (Davies et al. 2014; Souto et al. 2014). In our sample, outreach initiatives were only mentioned by 25% of respondents when defining conservation success. Although education and awareness initiatives have been employed, historically, amphibian conservation has not been linked to development projects that encompass livelihood provisions. However, this situation is changing (e.g., Bride et al. 2008; Lin et al. 2008) and is partly driven by donors increasingly supporting projects that also benefit people (e.g., Cunningham & King 2013). Consequently, outreach initiatives that benefit people are likely to become important outcome measures for future conservation interventions (Fisher et al. 2009).

Research and evaluation was related to success in terms of science‐based conservation practice. Improving the impact of conservation has been linked to the promotion of evidence‐based decision making (Pullin & Knight 2001; Sutherland et al. 2004) and the regular evaluation of outcomes (Bottrill & Pressey 2012). Although not frequently mentioned, research and evaluation is instrumental in achieving verifiable improvements in species and habitats. Furthermore, when rated against other components of a conservation program, research was second only to species and site management, indicating that it is of key concern in amphibian conservation (Table 1). The effects of conservation interventions can extend beyond project‐funding time scales (Kapos et al. 2008). The evaluation of short‐term and intermediate‐level success criteria may enable a project to progress stepwise toward long‐term impacts (Margoluis & Salafsky 1998; Margoluis et al. 2009; Martin et al. 2012). Additionally, measures of success may require ongoing negotiation between stakeholders, rather than be prescribed ex ante by external organizations (Sayer & Wells 2004).

### Predictors and Components of Success

Experience was a key predictor of perceptions of success in both academic and practitioner groups. More experienced practitioners tended to place greater emphasis on species and habitat improvements; less experienced practitioners tended to place more emphasis on outreach initiatives such as public education and engagement. Corresponding perceptions of nonpractitioners altered little with increased experience. Experience also influenced the importance attributed to human components of conservation. There was a trend for scores for education and awareness, sustainable resource use, and capacity building to be negatively associated with experience. More experienced practitioners may regard true success in terms of the traditional goal of effective management of species and habitats (Murphy 1990). Professional experience is often linked to career progression. This may draw perceptions of success away from program components and toward wider organizational goals or aspirations. Likewise, success from the ecosystem viewpoint can be displaced by internal priorities or self‐interest or disciplinary bias (Newing 2010; Sandbrook et al. 2011).

Although regarded as important, capacity building appeared to be the least popular component of success among our sample and was mentioned by only 1 respondent in the open‐answer question. However, it is increasingly emphasized globally as a key concern in promoting biodiversity conservation, for example in the Convention on Biological Diversity Aichi Biodiversity Targets under strategic goal E (CBD 2014). Capacity building can be achieved partly by bringing together local and international conservation practitioners and researchers, which helps strengthen local agencies to set and enact the conservation agenda (Knight & Cowling 2006; Smith et al. 2009). Capacity building may be seen currently (by practitioners and academics alike) as a wider organizational objective rather than as a project‐specific goal. If funding bodies set expectations that capacity building is achieved as part of a sustainable future for continued conservation achievement, it can become a key outcome and therefore a requirement within project design.

Research and evaluation was valued more by academics than practitioners, particularly for those involved in multiple programs. In academic institutions, career progression depends substantially on publishing (Sutherland et al. 2011), and this may explain the greater emphasis on research. Although it varies among organizations, publishing may be less of a priority for practitioners (Arlettaz et al. 2010). Respondents from LEDCs who were involved with more programs placed less emphasis on the importance of research. Wealthier countries view evidence‐based decision making as fundamental to success, whereas less‐wealthy countries may prioritize other actions out of socioeconomic need (Karlsson et al. 2007; Sunderland et al. 2009). Finally, importance scores for government policy were positively related to involvement in multiple programs for practitioners. For academics, the relationship was slightly negative. Effective policy and legislation are germane to the attainment of many conservation objectives (Rands et al. 2010; Phillis et al. 2013) and are important indicators of changes within a local or national conservation context, but they may be a low priority for academics (Arlettaz et al. 2010).

### Defining Success

Success is a value interpretation (Büscher 2014) shaped by worldviews (Jones 2012), which may be influenced by personal experiences, geographic location, and training. Clearly, understanding the determinants of success requires an assessment of both processes and outcomes, which are both measureable. The most fundamental outcome in conservation is recovery of the species, measured by population assessment. Indeed, measuring population recovery is the ultimate indication that other lower level outcomes have been achieved, such as mitigation of threats and restoration of habitat. Nevertheless, lower level processes and outcomes may require their own measureable indicators as checks and balances that programs are on track. For small, cryptic, and often highly seasonal species that display natural population fluctuations, determining population recovery can be difficult and take a long time (Keith et al. 2015). For amphibians—which have undergone rapid, catastrophic declines in some species and some regions—there may not be the time or resources to measure population recovery, and lower level, interim measures of success may be needed to monitor progress. Likewise, some of the processes—such as education and capacity building—may be inherently difficult to measure in terms of their outcomes. Establishing that education has worked and that capacity has been built does not necessarily translate into the behavior changes that may be needed to achieve threat mitigation and ultimately population recovery. Failure to see concrete evidence of these processes leading to positive outcomes may explain the perception of their low importance, particularly among more experienced conservationists. Coupled with the issue that amphibians are often overshadowed by mammals and birds when it comes to conservation campaigning, perhaps it is not surprising that the amphibian conservation community seems to be skeptical about the importance of capacity building. Equally, the fact that much conservation research fails to inform conservation practice may feed perceptions that research is not a major factor in driving success (Griffiths & Dos Santos 2012).

In amphibian conservation, there is a mismatch between the urgency for action and the time needed to implement well‐designed programs. Such programs require actions that tackle both the environmental and social drivers of declines and that identify measures of success. Consequently, amphibian conservation programs frequently focus on relatively low‐risk components that can be reasonably achieved within the time frame of a short‐term grant. The Amphibian Conservation Action Plan (Wren et al. 2015) provides the framework for joining up these components, identifying the gaps, and monitoring progress.

## Supporting information

The questionnaire (Appendix S1) and details of the explanatory variables (Appendix S2) are available online. The authors are solely responsible for the content and functionality of these materials. Queries (other than absence of the material) should be directed to the corresponding author.

Supporting Information
