# Supplementary material for: Practitioner and scientist perceptions of successful amphibian conservation
Source: Conserv Biol. 2018 Jan 10;32(2):366–75. doi: 10.1111/cobi.13005 (PMC6849735; doi:10.1111/cobi.13005)
Supplement: Supplementary file 1 — The questionnaire (Appendix S1) and details of the explanatory variables (Appendix S2) are available online. The authors are solely responsible for the content and functionality of these materials. Queries (other than absence of the material) should be directed to the corresponding author. [file COBI-32-366-s001.docx]

**Appendix S1: Questionnaire**

***Author note:*** *For the purposes of this manuscript, explanatory variables were derived from answers to questions 3,4,5,6 and 8. Definitions of success from the “open-ended question” were taken from answers to question 12. An exploration of “components of success” used the answers to question 15.*

1. What is your name? (Including title) …………………………………………………………………….
2. What is your nationality? …………………………………………………………………………….........
3. Who is your employer? ……………………………………………………………………………………
4. What is your job title? ……………………………………………………………………………………...
5. Which of the following best applies to you? (Tick more than one option if necessary)

□ Amphibian scientist / researcher

□ Amphibian conservation practitioner

□ Scientist / researcher (other) – please specify subject area…………………..…………….……..

□ Conservation practitioner – please specify taxa / location(s)

………………………………………………………………………………………………………….……..

□ Other – (please specify any role that links you to amphibian conservation / research)

………………………………………………………………………………………………………….……..

1. How many years have you worked in *amphibian* research / conservation practice? …………………………………………..
2. What has been the main focus of your research or conservation practice over the last 5 years?

………………………………………………………………………………………………………….………….....………………………………………………………………………………………………….………………….

**Conservation programme:** A planned and coordinated initiative designed to improve the status of a species, habitat and/or ecosystem through targeted conservation interventions.

**Conservation intervention:** Any activity that is carried out with the aim of facilitating and/or promoting the conservation of a species / habitat / ecosystem.

1. How many *amphibian* conservation programmes are you currently involved in?

Please provide actual number ……………………….

1. Please provide basic details of any amphibian conservation programmes are you currently involved in:

………………………………………………………………………………………………………………………

………………………………………………………………………………………………………………………

1. Please list up to 5 amphibian conservation programmes that **you believe** to be ***successful*** – where possible, please write the name of the species and programme concerned (if you know of none, please write ‘none’)

1.)…………………………………………………………………………………………………………………...

2.)…………………………………………………………………………………………………………………...

3.)…………………………………………………………………………………………………………………...

4.)…………………………………………………………………………………………………………………...

5.)…………………………………………………………………………………………………………………...

1. Please list up to 5 amphibian conservation programmes that **you believe** to be ***unsuccessful*** – where possible, please write the name of the species and programme concerned (if you know of none, please write ‘none’)

1.)…………………………………………………………………………………………………………………...

2.)…………………………………………………………………………………………………………………...

3.)…………………………………………………………………………………………………………………...

4.)…………………………………………………………………………………………………………………...

5.)…………………………………………………………………………………………………………………...

1. How do you perceive “success” in a conservation programme? Please write briefly about what success means to you in the context of a conservation programme.

………………………………………………………………………………………………………….…………...

………………………………………………………………………………………………………….…………...

………………………………………………………………………………………………………….…………...

………………………………………………………………………………………………………….…………...

………………………………………………………………………………………………………….…………...

**Conservation research:** The study of a species / habitat / ecosystem for conservation science purposes with the intention of publication in the peer-reviewed literature.

**Conservation evidence:** Scientific research that seeks to evaluate the degree to which a conservation intervention is effective at reducing threats to a species / habitat / ecosystem, or promotes the ability of a target species / habitat / ecosystem to resist or respond to those threats.

1. In your opinion, which areas of conservation research are most important for the successful *practice of conservation*? Please select your **TOP 5** choices.

| **Conservation research categories** | **Most important for the successful *practice of conservation*:**  Please select your TOP 5 choices |  |
| --- | --- | --- |
| Baseline data (assessing species presence and distribution) |  |  |
| Study of species biology / ecology |  |  |
| Long-term species monitoring |  |  |
| Population Viability Analysis |  |  |
| Population genetics |  |  |
| Species systematics |  |  |
| Captive breeding |  |  |
| Threat processes |  |  |
| Developing management strategies |  |  |
| Analyses of the overall performance of conservation programmes |  |  |
| Conservation evidence |  |  |
| Prioritising species or conservation areas |  |  |
| *Other:*  ***Please specify*** | *(i.)* |  |
|  | *(ii.)* |  |
|  | *(iii.)* |  |

1. Please read the following statements and score each statement on a scale of **'Highly important'** to **'Not important'** in terms of which best describe success in conservation, in your opinion. Tick any irrelevant statements as ‘Not Applicable (**N/A**)’.

Please then *select* your **TOP 3** statements (final column) according to which best describe success in conservation overall, in your opinion.

|  | **Statement** | **N/A** | **SCALE**  **Highly Not important important** | | | | | *Please* select *your* ***TOP 3*** *choices* |
| --- | --- | --- | --- | --- | --- | --- | --- | --- |
|  |  |  | **a** | **b** | **c** | **d** | **e** |  |
| **1.** | *Increasing the likelihood of persistence of native ecosystems, habitats, species and/or populations in the wild without adverse effects on human well-being* |  | **a** | **b** | **c** | **d** | **e** |  |
| **2.** | *Long-term persistence of reintroduced population(s) of conservation target species* |  | **a** | **b** | **c** | **d** | **e** |  |
| **3.** | *Wild population of conservation target species is stable or increasing, as indicated by appropriate monitoring and evaluation* |  | **a** | **b** | **c** | **d** | **e** |  |
| **4.** | *The status of the conservation target species has been downgraded on the IUCN Red List of Threatened Species (e.g. from CR to EN)* |  | **a** | **b** | **c** | **d** | **e** |  |
| **5.** | *The status of the conservation target species has been downgraded by national level government relevant to the range area* |  | **a** | **b** | **c** | **d** | **e** |  |
| **6.** | *Defined conservation project goals have been achieved through measurable indicators* |  | **a** | **b** | **c** | **d** | **e** |  |
| **7.** | *Conservation target species is declining at a slower rate than before conservation interventions were initiated* |  | **a** | **b** | **c** | **d** | **e** |  |
| **8.** | *The reduction of the intensity of conservation actions over time as the outcomes of these actions have been effective, and become less significant to the overall survival of the species* |  | **a** | **b** | **c** | **d** | **e** |  |

1. Please read the following statements and score each statement on a scale of **'Highly important'** to **'Not important'** in terms of which actions are most significant in bringing about success in a conservation programme, in your opinion. Tick any irrelevant statements as ‘Not Applicable (**N/A**)’.

Please then *select* your **TOP 3** statements (final column) according to which are most significant overall in bringing about success in a conservation programme, in your opinion.

|  | **Statement** | **N/A** | **SCALE**  **Highly Not important important** | | | | | *Please* select *your* ***TOP 3*** *choices* |
| --- | --- | --- | --- | --- | --- | --- | --- | --- |
|  |  |  | **a** | **b** | **c** | **d** | **e** |  |
| **1.** | *Reducing known threats to improve the response of conservation target species to conservation interventions* |  | **a** | **b** | **c** | **d** | **e** |  |
| **2.** | *Promoting sustainable resource use and minimising damaging practices by relevant stakeholders* |  | **a** | **b** | **c** | **d** | **e** |  |
| **3.** | *Implementing relevant policies and/or promoting legislation relevant to conservation aims* |  | **a** | **b** | **c** | **d** | **e** |  |
| **4.** | *Increasing the quality and/or quantity of conservation action(s) through appropriate capacity building (training of project staff)* |  | **a** | **b** | **c** | **d** | **e** |  |
| **5.** | *Increasing support for the conservation of a species among appropriate target audience(s) through a communication, education and public awareness strategy* |  | **a** | **b** | **c** | **d** | **e** |  |
| **6.** | *Applying appropriate research results to conservation practice* |  | **a** | **b** | **c** | **d** | **e** |  |

1. Please read the following statements and score each statement on a scale of **'Highly important'** to **'Not important'** in terms of which would best facilitate success in a conservation programme, in your opinion. Tick any irrelevant statements as ‘Not Applicable (**N/A**)’.

|  | **Statement** | **N/A** | **SCALE**  **Highly Not important important** | | | | |
| --- | --- | --- | --- | --- | --- | --- | --- |
|  |  |  | **a** | **b** | **c** | **d** | **e** |
| **1.** | *Leaders understand the project in the context of their role as a conservation leader* |  | **a** | **b** | **c** | **d** | **e** |
| **2.** | *Leaders provide people with clear direction and priorities that are relevant to conservation* |  | **a** | **b** | **c** | **d** | **e** |
| **3.** | *Leaders encourage discussion of difficulties and technical problems* |  | **a** | **b** | **c** | **d** | **e** |
| **4.** | *Leaders encourage the flow of ideas/opinions, both up and down the hierarchy* |  | **a** | **b** | **c** | **d** | **e** |
| **5.** | *Plans are adapted if approaches are ineffective or circumstances / project priorities change* |  | **a** | **b** | **c** | **d** | **e** |
| **6.** | *Goals are set on the basis of conservation needs and not on arbitrary aspirations* |  | **a** | **b** | **c** | **d** | **e** |
| **7.** | *Goals are established from knowledge or reasonable assumptions* |  | **a** | **b** | **c** | **d** | **e** |
| **8.** | *Plans and priorities are communicated clearly to people working on the project* |  | **a** | **b** | **c** | **d** | **e** |
| **9.** | *Plans are used to guide the work undertaken by colleagues and partner organisations* |  | **a** | **b** | **c** | **d** | **e** |

1. Please read the following statements and score each statement on a scale of **'Highly important'** to **'Not important'** in terms of which would best facilitate success in a conservation programme, in your opinion. Tick any irrelevant statements as ‘Not Applicable (**N/A**)’.

|  | **Statement** | **N/A** | **SCALE**  **Highly Not important important** | | | | |
| --- | --- | --- | --- | --- | --- | --- | --- |
|  |  |  | **a** | **b** | **c** | **d** | **e** |
| **1.** | *People are selected, trained & given roles that suit their skills, capability & team contribution* |  | **a** | **b** | **c** | **d** | **e** |
| **2.** | *People are given authority to make decisions and implement action according to their ability and close proximity to the work* |  | **a** | **b** | **c** | **d** | **e** |
| **3.** | *Funding is accessed to meet priorities, rather than funding itself determining what work should be done* |  | **a** | **b** | **c** | **d** | **e** |
| **4.** | *Conservation results are actively investigated and measured to establish effectiveness* |  | **a** | **b** | **c** | **d** | **e** |
| **5.** | *Conservation results are analysed to inform future decision-making and action* |  | **a** | **b** | **c** | **d** | **e** |
| **6.** | *Funding & resources are allocated to meet the needs of conservation work ‘on the ground’* |  | **a** | **b** | **c** | **d** | **e** |
| **7.** | *Funding and project resources are allocated in a timely manner* |  | **a** | **b** | **c** | **d** | **e** |
| **8.** | *Conservation work and decision-making is informed by scientific knowledge and where possible scientific, social or economic data* |  | **a** | **b** | **c** | **d** | **e** |

1. Please *select* your **TOP 5** statements according to which are most important overall to the success of a conservation programme, in your opinion.

|  | **Statement** | *Please select*  ***TOP 5***  *choices* |
| --- | --- | --- |
|  |  |  |
| **1.** | *Leaders understand the project in the context of their role as a conservation leader* |  |
| **2.** | *Leaders provide people with clear direction and priorities that are relevant to conservation* |  |
| **3.** | *People are selected, trained & given roles that suit their skills, capability & team contribution* |  |
| **4.** | *Leaders encourage discussion of difficulties and technical problems* |  |
| **5.** | *People are given authority to make decisions and implement action according to their ability and close proximity to the work* |  |
| **6.** | *Leaders encourage the flow of ideas/opinions, both up and down the hierarchy* |  |
| **7.** | *Funds are accessed to meet priorities, rather than funding itself setting the agenda* |  |
| **8.** | *Plans are adapted if approaches are ineffective or circumstances / project priorities change* |  |
| **9.** | *Goals are set on the basis of conservation needs and not on arbitrary aspirations* |  |
| **10.** | *Goals are established from knowledge or reasonable assumptions* |  |
| **11.** | *Conservation results are actively investigated and measured to establish effectiveness* |  |
| **12.** | *Conservation results are analysed to inform future decision-making and action* |  |
| **13.** | *Funding & resources are allocated to meet the needs of conservation work ‘on the ground’* |  |
| **14.** | *Funding and project resources are allocated in a timely manner* |  |
| **15.** | *Conservation work and decision-making is informed by scientific knowledge and scientific, social or economic data (where possible)* |  |
| **16.** | *Plans and priorities are communicated clearly to people working on the project* |  |
| **17.** | *Plans are used to guide the work undertaken by colleagues and partner organisations* |  |

**Thank you for taking the time to participate in this questionnaire!**

I am currently selecting a sample of amphibian conservation programmes to evaluate. If you are willing to be contacted about this in the future, please provide your contact details:

**…………………………………………………………………………………………………………………………………………………**
