# Supplementary material for: Practitioner and scientist perceptions of successful amphibian conservation
Source: Conserv Biol. 2018 Jan 10;32(2):366–75. doi: 10.1111/cobi.13005 (PMC6849735; doi:10.1111/cobi.13005)
Supplement: Supplementary file 2 — Supporting Information [file COBI-32-366-s002.docx]

**Appendix S2: Explanatory variables and open-answer question point categories**

**(i) Explanatory Variables**

**Table S1.** Information collected from respondents forming the basis of five potential explanatory variables

| **Explanatory variables** | **Type** | **Definition** |
| --- | --- | --- |
| **Institution** | Categorical Levels: Academic; Non-academic | ***Employer and/or institutional base of respondent***  Academic institutions: any organisation with the principle objective of research and contributing to peer-reviewed literature, e.g. universities, research institutes, research-led museums.  Non-academic institutions: any organisation with non-academic principle remits associated with amphibian-related matters, such as conservation practice, policy, husbandry, non-research based awareness-raising activities e.g. NGOs; government agencies, zoos and aquaria. |
| **Country** | Categorical Levels: MEDC; LEDC | ***Location of respondent’s employer and/or institution***  Countries are listed as a More Economically Developed Country (MEDC) or a Less Economically Developed Country (LEDC) by the International Monetary Fund (i.e. Advanced and Developing Economy countries (IMF 2014). |
| **Practitioner** | Categorical Levels: Conservation Practitioner - Yes; No | ***Respondents asked to specify whether they described themselves as a 'conservation practitioner'***  "Conservation practitioner" describes anyone with active practical involvement (i.e. aside from research) in conservation programme(s) - defined in this questionnaire as: "*a planned and coordinated initiative designed to improve the status of a species, habitat and/or ecosystem through targeted conservation interventions*". A practitioner may or may not also be a conservation scientist/researcher. |
| **Experience** | Continuous (in years) | **The number of years the respondent has worked in amphibian research and/or conservation practice.** |
| **Programmes** | Continuous (number of programmes) | **The number of operational conservation projects currently undertaken by the respondent as a participant/partner.** |

**(ii) Point categories and counts for open answer question: “*How do you perceive success in a conservation programme?*”**

**Table S2.** Species & Habitat: point categories and counts

| **Response Variable** | **Sub category** | **Point types** | **How do you perceive 'success' in conservation? Example quotes** | **Total number of points across all respondents** |
| --- | --- | --- | --- | --- |
| **Species & Habitat** | **In situ conservation actions** | Wild population improvement  (numbers, persistence) | *“Success would be turning negative population trends into positive or stable population trends”  "Persistence of the population, together with links to the wider countryside, successful recruitment and continued expansion of their range"* | 200 |
|  |  | Habitat improvement (condition, size, connectivity, protection) | *“The habitat is protected and intact enough to allow the species to exist on an ongoing basis”* | 72 |
|  |  | Threat mitigation | *“A resilient target species, community or ecological function by the reduction of key threats”* | 30 |
|  |  | Wild population re-establishment | *“Successful re-establishment of a threatened species in the wild, including wild reproduction into the second generation”* | 18 |
|  |  | Wild population condition  (e.g. genetic diversity, health) | *“Succeeding in obtaining a system that maintains population dynamics, demographic numbers and genetic diversity”* | 9 |
|  |  | Species status improvement | *“Long term survival of the target species illustrated through potential downlisting of the species on the IUCN Red List”* | 6 |
|  | **Ex situ conservation actions** | Captive breeding and genome banking for conservation purposes | *“For species where the threats cannot be mitigated in time to save the species, a successful conservation program involves bringing a large enough number of founders into captivity within the range country, to establish an ex situ assurance population in biosecure facilities while the threats are mitigated, and then releasing captive-bred animals back into the wild”  "Safe-guarding biodiversity - not just recovery in situ but also ex situ population and genome resource banking for the future"* | 14 |

**Table S3.** Programme Management: point categories and counts

| **Response Variable** | **Sub category** | **Point types** | **How do you perceive 'success' in conservation?  Example quotes** | **Total number of points across all respondents** |
| --- | --- | --- | --- | --- |
| **Programme Management** | **General programme structure, timeframe and management** | General programme structure and management | *“The programme is well thought-out, addresses all necessary components and is being undertaken in an adaptive manner”  “Successful development of the programme, including personnel, funding and activities”*  *"Using an adaptive management and learning programme framework – increasing knowledge and changing programme to accommodate change”* | 39 |
|  |  | Programme has reached a point where interventions can be reduced or are no longer required | *“Top level success would be where there are sustainable populations in the wild with the minimum amount of actions or no further actions needed at all by conservation practitioners”* | 14 |
|  |  | Programme timeframe (i.e. long-term programme action) | *“Success in a conservation programme is a long term goal and it cannot be determined over a short period”* | 13 |
|  |  | There are different types/degrees/stages of success that must be managed | *“Success is a very broad term when dealing with the conservation of amphibians. There are so many different factors and influences. Success is often gauged in steps rather than final outcomes”* | 13 |
|  |  | Policy and legislation | *“The mindset of the government is such that the programme will continue”* | 8 |
|  | **Achievement of stated programme goals** | Programme outcome achievements: goals and objectives | *“As a trained project manager I think success is achieving the stated goals. But I must admit that the goals are often not 1 on 1 with real conservation”*  *"* *To me the success of a project relates to the aim and objectives for that particular project, which should be specific, objective, and within a certain timeframe. I think many projects fail to have clear objectives, and therefore it is difficult to establish whether or not they have been a success"* | 25 |

**Table S4. Education & Engagement and Research & Evaluation**: point categories and counts

| **Response Variable** | **Sub category** | **Point types** | **How do you perceive 'success' in conservation?  Example quotes** | **Total number of points across all respondents** |
| --- | --- | --- | --- | --- |
| **Education & Engagement** | **Public education & awareness activities** | Public education and awareness activities | *“Engage in educating people to be aware of the reasons for decline of native amphibian populations and ways they can make a positive difference in protecting the native species in their localities”  “All ‘successful’ programs need to have outreach components in order for them to be truly successful in the long run”* | 44 |
|  | **Local community/ stakeholder support & involvement** | Fostering local community/stakeholder involvement | *“Programmes that save wild places (not just wild animals) seem to have the greatest impact when there is local community involvement and support”* | 21 |
|  |  | Benefiting local stakeholders and/or gaining their support  Capacity Building | *“Every program will be successful if the focus is on people, especially in developing and poor nations. The general public will not show interest in the project until it is directly linked with the sustenance of the people”*  *“Capacity that is built within the region through involvement of local agencies”* | 12  1 |
| **Research & Evaluation** | **Species/habitat scientific research** | Species/habitat scientific research | *"Filling knowledge or data gaps that contribute indirectly to conservation”  "A solid understanding of the species biology and disease risk is vital"  "Basic research that informs any aspect of amphibian conservation or biodiversity or natural history should be considered as a success"* | 25 |
|  | **Evaluation of programme outcomes through appropriate monitoring** | Evaluation of programme outcomes through appropriate monitoring | *"A program that is executed efficiently and that involves multiple agencies to implement conservation results as well as monitoring or evaluating successes"  “Success comes with a lot of population monitoring and going out of your own way to save that species”* | 15 |


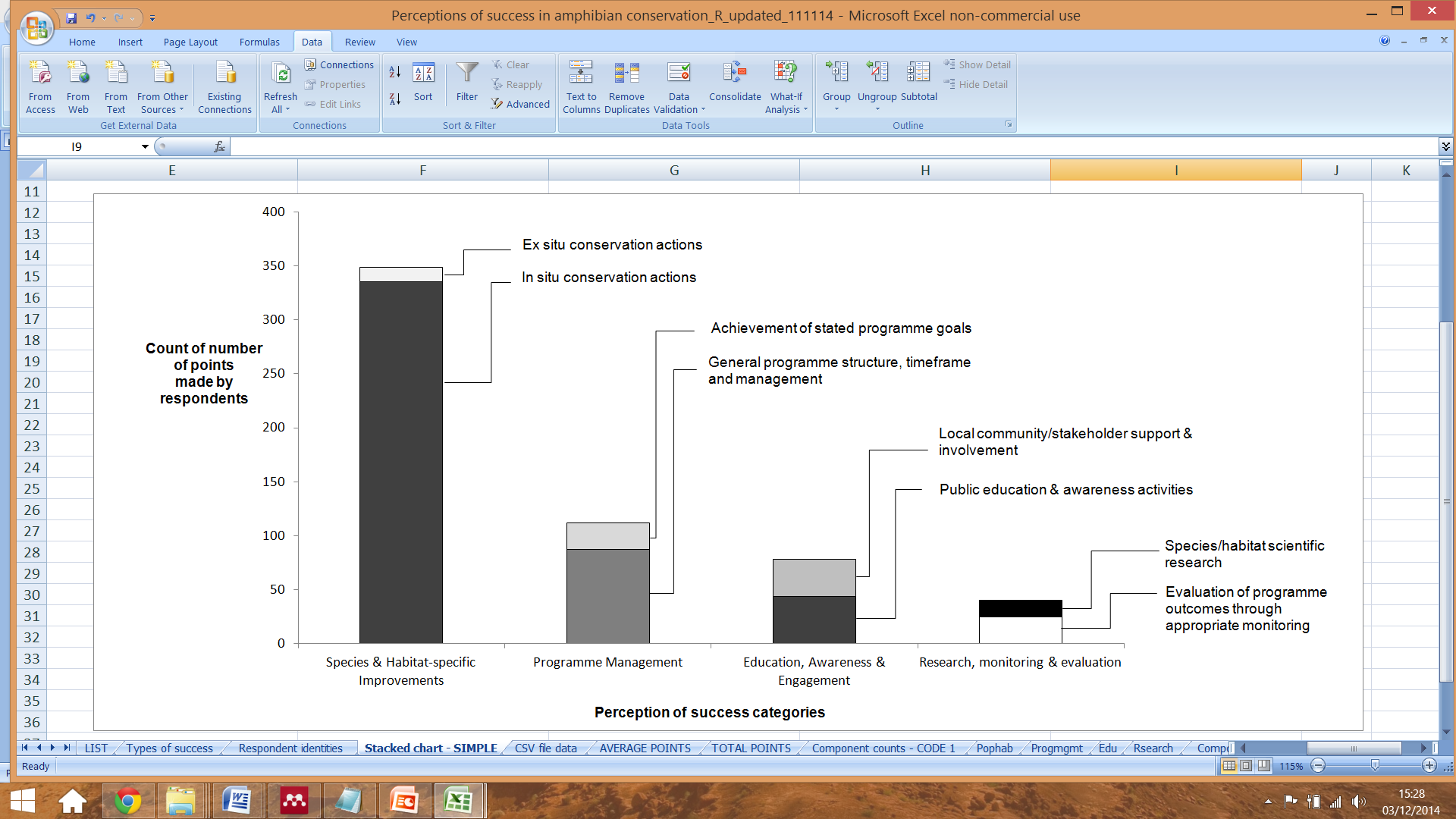


**Fig. S1** Stacked column chart of categories (and sub-categories) displaying the number of points made in response to the question “*How do you perceive ‘success’ in a conservation programme? Please write briefly about what success means to you in the context of a conservation programme*” (n = 242 respondents/579 discrete response points).
